# The crucial decade that ion channels were proven to exist: The vision of Bertil Hille and Clay Armstrong and how it came through

**DOI:** 10.1007/s00424-025-03085-5

**Published:** 2025-04-22

**Authors:** Luigi Catacuzzeno, Antonio Michelucci, Fabio Franciolini

**Affiliations:** Dipartimento di Chimica Biologia e Biotecnologie, Universita’ di Perugia, Perugia, Italy

**Keywords:** Cell excitability, Ion channels, Retrospective on ion channels

## Abstract

This retrospective begins with the first recording of the Na^+^ and K^+^ currents underlying the action potential in the squid giant axon reported by Hodgkin and Huxley in 1952, which made the question of where ions pass through the membrane more compelling. The notion of channels in the membrane had been around for quite some time but was so vague and contested that even the recording of Na^+^ and K^+^ currents through the membrane was not considered sufficient proof of their existence. In fact, Hodgkin and Huxley never referred to ion channels in their papers, only currents and conductances. The word “channel” remained somewhat left out from the scientific debate for almost another two decades, even though its idea was slowly making its way into the minds of discerning scientists. It is precisely this period that the present retrospective focuses on to understand the evolution of the ion channel concept from a speculative functional entity to a physical transmembrane object that serves the efficient and selective passage of ions. In this regard, the fundamental contribution of Bertil Hille and Clay Armstrong in promoting this idea, in the cold attitude, when not open aversion, of much of the scientific community, is fully acknowledged. Mention should also be made of Erwin Neher and Bert Sakmann’s patch-clamp technique, which made it possible to directly measure ion currents through individual channels, thus conclusively demonstrating their presence in cell membranes. The retrospective goes on to briefly show how the cloning of ion channels in the 1980s and the first X-ray crystallographic structures at the turn of the century fully confirmed the initial suggestions, and closes by illustrating the relevance of ion channels in biology and medicine.

## Introduction

Ion channels are integral membrane proteins with a central pore for the passage of ions. While some channels poorly select the ion species to be let through, others do so remarkably well. It is on their high selectivity to specific ions that many different channels have been named, such as the voltage-gated Na^+^ channels, K^+^ channels, and Ca^2+^ channels. By contrast, agonist-gated channels, such as those activated by neurotransmitters, are normally poorly selective and often let Na^+^ and K^+^, and Ca^2+^ at times, pass just as easily. A typical channel protein can mediate the passive transfer of millions of ions per second across the membrane, with the flux occurring under the combined action of the concentration gradient and the electric field (the transmembrane potential), according to the Nernst relation of the electrochemical potential.

The channel pores are not always open but are regulated to switch by various electrical, chemical, or physical inputs from the environment. It was based on the type of stimulus they respond to that the channels have been classified as voltage-gated channels, such as the Na^+^ and K^+^ channels underlying the action potential of many excitable cells, agonist-gated channels, such as the postsynaptic receptor channels activated by neurotransmitters, or, for instance, mechanosensitive channels opened by membrane stretch.

In the early 1980s, several channel proteins were cloned, their amino acid sequences determined, and the first channel structures proposed. These were refined, only a few years later, when the actual structures of several channels began to appear at atomic resolution from X-ray crystallography and later cryo-EM. MD simulations and other computational and theoretical approaches that followed shortly thereafter on the shoulders of these high-resolution structures have contributed enormously to better defining most aspects of both ion permeation and channel gating to the point where we can now say that we know a great deal about them.

However, there was a time, not so long ago, when the very idea of the presence of ion channels in the membrane was viewed with deep skepticism by the scientific community. It was not at issue that ions crossed the membrane, but certainly how this could happen. This is despite the fact that the idea of pores (*kanäle*) in membranes is rather old, as it can be traced back to the 1840 s, when Brücke [13] proposed them to explain the osmotic processes he was studying in pig bladder. This notion was widely accepted in those years among leading German biophysicists such as Hermann von Helmholtz, Carl Ludwig, Emil DuBois Reymond, and Adolf Fick. The consensus was that these pores would allow water to pass along with very small particles, although no size estimates were given. This notion of “pores” circulated throughout much of the second half of the nineteenth century in both scientific discussions and teaching lectures, although it remained mostly associated with osmotic processes at the organ level, as glomerular filtration. It was not yet accepted for cell membranes. Not a few at the time thought more of indefinite passages of ions through cracks in the cell membrane’s lipoid matrix, of transporters shuttling ions across, not to mention the few who even doubted the very existence of the membrane; they would rather think of a hardening of the outermost portion of the cytoplasm as the cell’s boundary structure. The question came up again, and this time with greater intensity, after Hodgkin and Huxley’s first recordings of the Na^+^ and K^+^ currents underlying the action potential of the squid giant axon in the early 1950s.

Over the following couple of decades, the debate concentrated on the hypothetical membrane structures for the passage of these currents. In recounting the facts and the people involved, we focus on the voltage-gated channels because they have been at the center of the main studies that have driven the evolution of the channel concept from a theoretical notion to a concrete protein structure in the membrane for the passage of ions.

## Hodgkin and Huxley’s time

In 1952, five papers by Hodgkin and Huxley appeared in *The Journal of Physiology* that explained the origins of the action potential, its propagation, threshold, refractoriness [46–50]. Today, these papers and their authors have achieved something like legendary status, certainly not only for their clear elucidation of a very important and complex biological phenomenon but also for the skill they demonstrated in extracting so much information from an objectively limited number of clearly designed experiments, for their use of the mathematics required to tackle difficult experimental problems, and for their creativity and imagination in interpreting complex results. They dissected and quantitatively described the Na^+^ and K^+^ currents of the squid giant axon by an empirical kinetic model of voltage- and time-dependent changes in ion permeability, which accurately reproduced (simulated) the generation and propagation of the action potential. Their description was purely mathematical, on an electrical framework, without any commitment to the nature of the structures underlying the changes in membrane permeability; in other words, no reference was ever made to ion channels in any of their papers. Hodgkin and Huxley’s work opened a new era in channel biophysics, inspired generations of membrane biophysicists and still does today, 70 years after its publication, and earned its authors the Nobel Prize in Physiology or Medicine in 1963 (for a recent review, see Catacuzzeno & Franciolini, [15]).

### The first recording of the Na^+^ and K^+^ currents

In the summer of 1949, Hodgkin and Huxley, building on the experience of the previous experimental season, performed all the voltage clamp experiments and recorded all the currents that they used to describe and interpret the ionic basis of the action potential and its propagation that appeared in those “legendary” papers. The recorded currents had a complex time course, with an initial inward phase followed by an outward opposite phase. Based on the Nernst equation, the inward and outward current components could be assumed to be due to Na^+^ entry into the axon and K^+^ exit, respectively. They verified this by repeating the experiments after replacing the external Na^+^ with impermeable choline, which eliminated the inward component, leaving only the outward current, arguably the isolated K^+^ current. To obtain the putative Na^+^ current, they simply subtracted the outward K^+^ current from the total current, assuming that these were the only two currents present, which turned out to be essentially correct for the squid giant axon.

We wish to recall, in Hodgkin’s words, how in the summer of 1949, in about a month, they managed to complete all the experiments used in the five papers published in 1952, as a special lesson for today’s times, when everything seems to move so fast and often with little thought behind it: “I think that we were able to do this so quickly and without leaving too many gaps because we had spent so long thinking and making calculations about the kind of system which might produce an action potential of the kind seen in squid nerve. We also knew what we had to measure in order to reconstruct an action potential.”

From experiments of this type, conducted at different clamped voltages and using varying protocols, Hodgkin and Huxley concluded that Na^+^ and K^+^ currents result from separate and independent permeability processes that depend on time and membrane potential (the ionic hypothesis of the action potential). On those data, they developed a refined kinetic description that correctly predicted the main properties of membrane excitability, such as the time course of the action potential, the firing threshold, the shape of the Na^+^ and K^+^ currents, and their voltage dependence. Although subsequent experiments revealed several specific inconsistencies, the model is regarded as a breakthrough in membrane biophysics.

To account for the sigmoidal rise of Na^+^ and K^+^ conductance, Hodgkin and Huxley postulated that separate pathways were formed for the two ions when a cluster of independently moving charged particles associated with each pathway moved across the membrane from a “non-permissive” into a “permissive” position (Hodgkin and Huxley terminology) upon depolarization. For the Na^+^ passage, they found that the data were well fitted when three charged particles were made to move to the “permissive” position to open its pathway, while four charged particles had to move to open the membrane pathway to K^+^. The inactivation of the Na^+^ current was accounted for by the translocation of single charged particles that blocked the ion passage. These activation gating particles that control the pathways of Na^+^ and K^+^ ions through the membrane would move back to the “non-permissive” position upon repolarization, the difference being that it only takes one of them to return to the “non-permissive” position for the pathway to close off the passage of ions.

An inescapable corollary of this activation model, which Hodgkin and Huxley recognized immediately, is that the postulated charged particles moving across the membrane in a voltage-dependent manner should generate small transient currents (the small size of the current was assumed based on other experimental data they produced on the side). They actually tried to record them, but without success, which led them to believe that if these currents were ever recorded, they would have to be very small, hardly more than a few percent of the ionic currents. Some 20 years later, these currents were indeed recorded [7, 96]. They were called “gating currents” because of their supposed association with the gating process and were indeed about 2% of the peak Na^+^ current.

Notably, in none of their papers did Hodgkin and Huxley ever mention “ion channels,” only ion currents and conductance. In fact, the concept of an ion channel, as we know it today, did not even exist at the time. Carriers were more in vogue in the scientific community, also in association with membrane excitation. In the last of their 1952 papers, commenting on the Na^+^ inward current, Hodgkin and Huxley [49] wrote that it could not be excluded “the possibility that Na^+^ ions cross the membrane in combination with a lipoid solubile carrier.” This shows how strongly rooted the concept of the carrier was at the time, and how far removed the concept of the ion channel was.

## Ion channels: from functional concept to molecular entity

The concept of the ion channel did not come about abruptly but matured over a whole decade at least—a decade of discussions, mismatched experimental interpretations, and lively debates. When the lipid membrane was proposed at the turn of the century [65, 83], its failure to explain the high permeability to water was overcome by suggesting that the lipid layer contained pores that would allow not only water but also ions to pass through [93]. Michaelis [66] went further, suggesting that ions passed through these holes by adsorbing to their walls. This view of the ion-pore interaction gained more traction when, 30 years later, it was used by Hodgkin and Keynes [51] as a possible explanation for the results of the K^+^ isotope flux experiments. During a careful investigation on the squid giant axon to see how K^+^ ions would cross the membrane, they unexpectedly found that the influx of isotopic K^+^ at constant membrane potential did not increase linearly but exponentially with the external K^+^ concentration, showing, along with other clues of similar tenor, that K^+^ ions do not cross the membrane independently [51]. In an attempt to explain this “anomalous”—in the sense of unexpected—K^+^ flux, after considering several mechanisms, they seemed to favor the one in which, in their own words, “ions cross the membrane along a chain of negative charges or through narrow tubes or channels… in which they are constrained to move in single file.” The concept of pores was expressed again by Arthur Solomon in his [103] *Scientific American* review “Pores in the cell membrane” to explain the different permeability to cations and anions across the membrane.

### The birth of a concept

In those days, the terms “pore” and “channel” were used as synonyms without anyone objecting, as they were only seen as lexical tools to indicate undefined holes in the membrane for the passage of ions, without any mechanistic implications. The term “ion channel” was initially used in reference to Na^+^ [29, 52] and K^+^ [45]. The term was also used by Katz [55] in a review on synaptic transmission, but this time, it was associated with an important property: ion selectivity. From just a lexical tool, the term “channel” abruptly evolved into a physical object capable of passing and selecting ions. It was suggested that to do this, ions had to dehydrate (even partially) to go through, which raised pressing energy questions due to the high cost of stripping water from them. To overcome this problem, Mullins [75] proposed that portions of the pore regions within the channel form ion cages or binding sites to facilitate ion dehydration by providing surrogate electrostatic interactors and consequent binding energy (also see Eisenmann [28]). These interaction sites within the pore help explain the ion selectivity exhibited by some channels, e.g., Na^+^ vs K^+^ channels [74, 75]. However, there was still reluctance to consider separate pores selectively passing specific ions (as opposed to a single pore letting Na^+^ pass first during the action potential rise and then K^+^ once the Na^+^ conductance had died down), even though it had already been shown that tetraethylammonium (TEA) blocked selectively the K^+^ current, i.e., without affecting the Na^+^ current [8, 109]. It took the discovery of tetrodotoxin (TTX), a potent neurotoxin commonly found in certain marine fish, which selectively blocked the Na^+^ current without any effect on the K^+^ current [76], to further consolidate the notion of separate pathways. Using this selective blocker of the Na^+^ current and the assumption that it would bind in a 1:1 ratio to the postulated Na^+^ channels, Moore [70] estimated that no more than 13 Na^+^ channels per square micron were present on the lobster nerve membrane. These data and earlier voltage clamp results that set an upper limit to 100 Na^+^ channels per square micron [64] suggested that Na^+^ permeation pathways in the membrane—i.e., Na^+^ channels—were in any case very sparsely dislocated.

**Fig. 1:**
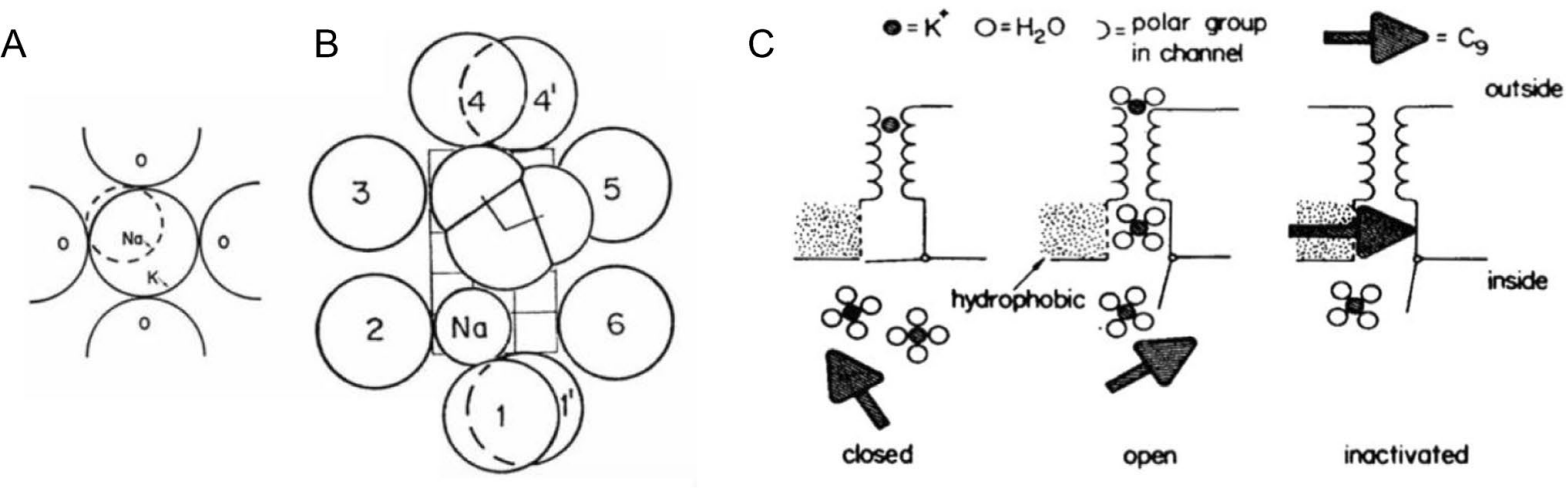
Early views of Na⁺ and K⁺ channel structure, permeation, and block. **A** Delayed rectifier K⁺ channel showing the four oxygens coming in close contact with a permeating K⁺ ion but not with a Na⁺ ion. This cartoon and that shown in **B** are drawn in proportion to the ions’ crystal radii (1.33 and 0.95 Å for K⁺ and Na⁺ ion, respectively (from Bezanilla and Armstrong [12]. **B** Voltage-gated Na⁺ channel with an estimated size of 3 × 5 Å (see grid in background) illustrates the coupled passage of a Na⁺ ion standing at one corner of the filter and a water molecule filling the remaining space formed by the eight oxygen dipoles of the Na⁺ pore (from Hille [36]). **C** Cartoon showing nonyl triethylammonium ion (C9^+^) block of the delayed rectifying K^+^ channel and the inferred mechanism of action. In the resting state, the channel is closed (left) and C9^+^ cannot bind to it. When the channel opens by depolarization, K^+^ ions begin to pass through (center), until the slowly diffusing C9^+^ enters the pore and blocks the passage (right). This mechanism whereby the channel conducts for a short time before being blocked or “inactivated” is perfectly consistent with the experimental observation of a transient outward K^+^ current generated by internal application of C9.^+^ (from Hille et al. [41])

Classic biophysical experiments beginning in the mid 1960s, which showed distinct conduction properties for different ions, began to provide the first clues as to the architecture and basic physico-chemical properties of the conduction pores and the mechanisms underlying ion permeation and selectivity. Hille focused his efforts on investigating the selectivity properties of these membrane pores with the idea that it would perhaps lead to something instructive regarding their structural and chemical properties. A few studies had already addressed this topic [64, 111] but not in a systematic way as Hille had in mind. By selectively blocking either pathway, his studies showed that at least 10 cations could easily permeate through the Na^+^ pores and four through the K^+^ pores of Ranvier’s node. Considering the size of the ions tested and the length of hydrogen bonds, Hille estimated the Na^+^ pores to have a size, in its most constricted portion, which he called the “selectivity filter,” of 3.1 × 5.1 Å and assumed to be lined by oxygen dipoles that would establish hydrogen bonds with the permeating cations (Fig. 1) [33, 36, 37]. Most interesting was another observation: all cations with a methyl group were impermeant, regardless of their size. In other words, large hydroxy guanidinium could go through the Na^+^ pore, while small methylammonium could not [12, 34, 36, 37]. These results provided experimental support for previous proposals that permeant ions interact with the pore wall and that this interaction contributes to the membrane’s permeation properties; in other words, the membrane or the pores in it do not merely select by ion size, as if they were simple physical sieves.

These studies also broke the long-standing paradigm in biology that ions permeate cell membranes in their hydrated form, a view based on experimental observations and theoretical considerations [26]. Firstly, the lack of correlation between the permeability coefficients of ions across the membrane and their atomic size, and secondly, the extremely high energy required to remove the hydration shell. In that decade, crystallographic studies were showing that ionophores such as valinomycin form selective binding sites that are highly complementary in size and coordination to specific ions, and flexible enough to adapt to the ion. Physico-chemical studies were also showing that these binding sites could selectively interact with dehydrated ions through hydrogen bonds, ion–dipole interactions, and electrostatic forces [69, 72]. In addition, the first models of hypothetical ion channels started appearing, which suggested that the channels could have hydrophilic regions to accommodate and bind dehydrated ions, allowing them to pass through the hydrophobic membrane in “naked” form [38]. These models seemed an effective answer to questions concerning the high-energy cost of ion dehydration and the lack of correlation between the permeability coefficients of ions and their atomic size. After all, how else could one explain that the Na^+^ pores can pass Na^+^, K^+^, amino-ammonium (hydrazinium), guanidinium, but not methylammonium, without considering the direct interactions between the pore walls (amino acid residues) and the naked ion as the only way for the pore to recognize details of the ion to decide whether or not to pass, as opposed to an ion covered by a multilayer water shell?

Other findings of that period, in particular Armstrong’s experiments with TEA^+^ derivatives on the outward K^+^ current of the squid giant axon, strengthened the notion that the membrane pores were at least partly made up of protein. Years earlier Tasaki and Hagiwara [109] had obtained action potentials with a long-lasting plateau, like the cardiac action potential, when they perfused internally the squid giant axon with TEA^+^. These data were interpreted as being due to a TEA^+^-dependent block of the outward K^+^ current (which they called anomalous rectification) and resulting failure of K^+^ current-dependent repolarization. Armstrong and Binstock [8] continued their investigation with TEA^+^ by probing the drug on the K^+^ current under voltage clamp, thinking that these compounds could disclose new mechanisms and the pore architecture. First, they found that internal TEA^+^ eliminated the outward K^+^ current, whereas it was totally ineffective when applied from the outside. However, the most interesting results came when Armstrong began probing a series of TEA^+^ derivatives made by replacing one of the four ethyl groups by a progressively longer hydrophobic chain and found that the efficacy of block increased with the chain length. Using C9^+^ (nonyl triethylammonium ion) from the inside, he found that the K^+^ current no longer reached a steady-state level during the voltage step but inactivated in a manner quite like the Na^+^ current [5].

These results are important for two reasons. Firstly, the mechanism of C9^+^-induced inactivation of the K^+^ current conclusively set aside the carrier model of ion transport across the membrane, since the results with C9^+^ could only be explained by a model pore, according to Armstrong. In short, K^+^ current inactivation by internally applied C9^+^ occurs when the drug binds with its long hydrocarbon chain to the internal portion of the pore, blocking the outward flux of K^+^ ions [5]. The observation that C9^+^ block can be removed more rapidly when the external K^+^ concentration or the hyperpolarizing step is increased is consistent with a membrane pore rather than a carrier. Secondly, the close similarity between C9^+^-induced inactivation of the outward K^+^ current and the inactivation of the Na^+^ current suggested to Armstrong the “ball-and-chain” model, according to which when the Na^+^ pore opens to allow Na^+^ ions to flow inward, the ball moves into the inner cavity of the pore from the cytoplasmic side to inactivate the channel. This interpretation was largely based on experiments with proteolytic enzymes showing that, applied from within the squid axon, they eliminated the inactivation of the Na^+^ current, presumably because the enzymes cut the “chain” and the channel lost the “ball” responsible for inactivation [7]. These results, among other things, reinforced the idea that these membrane pores must be made, at least in part, of proteins.

### The decisive contribution of Bertil Hille and Clay Armstrong

With the recognition of separate pathways for the passage of different ions (Na^+^ and K^+^) through the membrane and with the several other features described above in mind, a number of scientists began to systematically refer to them as Na^+^ channels and K^+^ channels, thinking of holes, pores, or a kind of small funnels in the membrane, with properties reminiscent of Mullins’ cages or binding sites [3–5, 8, 34–36, 64, 70]. In this crusade, Bertil Hille and Clay Armstrong were undoubtedly involved more deeply than anyone else. They were both convinced that Na^+^ and K^+^ ions use separate pathways to cross the membrane; in other words, they pass through different pores that should be called Na^+^ and K^+^ channels (for a first-hand retrospective on these topics, see Armstrong and Hille [9] and Hille et al. [41]). This view was not easy to accept, considering the scant supporting evidence and the strong skepticism of many scientists. The disagreement in this regard can best be illustrated by recalling the views of Ichiji Tasaki, an eminent membrane physicochemist and electrophysiologist in the 1960s and 1970s, who pioneered studies on saltatory conduction in mammalian myelinated fibers and the effects of internal TEA on the action potential in the squid giant axon. However, with regard to ion channels, he believed that membrane excitation could be explained on the basis of the two stable states of membrane macromolecules, which he had found experimentally, and the different physicochemical properties gained and lost with the phase transition between the two states. Namely, in the resting state, the negative sites on the outer surface of membrane macromolecules mainly bind external divalent cations (Ca^2+^), which in turn are replaced by monovalent cations (Na^+^) upon membrane excitation and transition of the membrane to the stable active state [57, 110]. In Tasaki’s view, membrane excitation could be explained without involving ion channels, which were instead much discussed at the time.

To further represent the general sentiment on the subject at that time, it may also be helpful to recount what happened at the 1966 Biophysical Society meeting, when Armstrong and Hille presented two separate abstracts, both with the word “channel” in the title. As Hille recalls in a recent retrospective [40] “the Chair of the session, Toshio Narahashi, began by announcing that the word ‘channel’ could not be used in the session. After our vigorous objection, he allowed us to use the word ‘provided it did not imply any mechanism!’”.

They were young but firmly convinced of their ideas and strongly determined to pursue them, even without the support from senior scientists or their mentors. On the contrary, on the other side of the ocean, someone was ready to give them and their ideas credit. In 1970, Hille was invited by Denis Noble of Oxford to write a review of the views on channels that he and Armstrong were pioneering. The title—“Ionic Channels in Nerve Membranes”—was decidedly disruptive. Once the final draft of the review was ready, Hille asked Kenneth Cole, Armstrong’s former mentor at NIH and a prominent biophysicist, to read it. After reading the draft, Cole sent him a note saying that he was “slightly concerned that you might be pushing some of the arguments in your channel rather far.” [40]. This harsh comment came as little surprise to Hille, who knew all too well Cole’s position on this issue.

The dispute on the presence of pores in the membrane went on for a while. Hille recalls of a paper with Wolfgang Schwarz submitted to the *Journal of General Physiology* in 1978 in which they used for the first time Eyring’s rate theory to describe ion permeability, assuming that ions passed through holes in the membrane [42]. The article had a difficult publishing course, as the editors felt the need to hear from a second round of referees before deciding whether to publish the article, as several channels were known to be finely modeled as carriers. This was one of the last times the notion of ion channels in the membrane aroused suspicion and concern and started to become mainstream thinking.

### Noise analysis and patch clamp provide final evidence of the presence of ion channels in cell membranes

It was surprising, however, that this subject still caused so much unease in 1978, given that at the time, convincing results had been provided in favor of ion channels in the membrane as individual entities with gating properties, which in the open state can pass millions of ions per second. Reference is first to the data from noise analysis of the macroscopic currents, pioneered by Verveen and Derksen [113] and by Katz and Miledi [56] (see also reviews by Stevens [104] and Neher and Stevens [78]), which provided evidence that the macroscopic currents now recorded in many cell types result from the contribution of many small unitary currents, arguably flowing through individual ion channels.

Using these methods, Conti et al. [21] found that the power spectral density of the current noise of the squid giant axon was consistent with the sum of two Lorentzian components, expected to be associated with the open–close kinetics of underlying K^+^ and Na^+^ channels. These data provided further evidence for the existence of ion channels underlying ion currents and membrane noise. Incidentally, the density of 330 Na^+^ channels per square micron on the membrane of the squid giant axon was also estimated in this study. The following year, using stationary fluctuation analysis, Robert Stämpfli et al. (including Wolfgang Nonner and Berthold Neumcke, from there, Bertil Hille on sabbatical leave from the University of Washington, and Conti from Italy) estimated the single-channel conductance of the Na^+^ channels of the node of Ranvier to be 7–9 pS [19]. When, years later, the single-channel conductance of the Na^+^ channel could be measured directly with the patch clamp, it was found to be very close to those values estimated indirectly by stationary fluctuation analysis.

The next goal was to test whether the gating charges moved in packets (quantal motion) upon channel activation, given the presence of four putative voltage sensors per channel. For this purpose, Conti and Stühmer [20] applied noise analysis to macroscopic gating currents (after developing the mathematical background, as this approach had never been tried before). Autocorrelation and variance analysis of gating current fluctuations, recorded from macropatches of *Xenopus* oocytes injected with mRNA coding for rat brain Na^+^ channels, suggested a shot-like movement of the gating charge upon Na^+^ channel activation, occurring in 2 to 3 brief packets, each carrying an equivalent of about 2.3 electron charges. Similar results were later obtained by Bezanilla and coworkers on expressed Shaker K^+^ channel [101]. Although neither of these results accounted perfectly for the total amount of gating charge moved during channel activation (estimated by different methods to be at least 10 for Na^+^ and K^+^ channels [1, 43, 97, 98, 122]), they strengthened the view of the discrete behavior of the gating charge movement, consistent with the multiplicity of voltage sensors present within these channels. A thorough discussion of the cause for the above inconsistency is contained in a recent review [14].

**Fig. 2:**
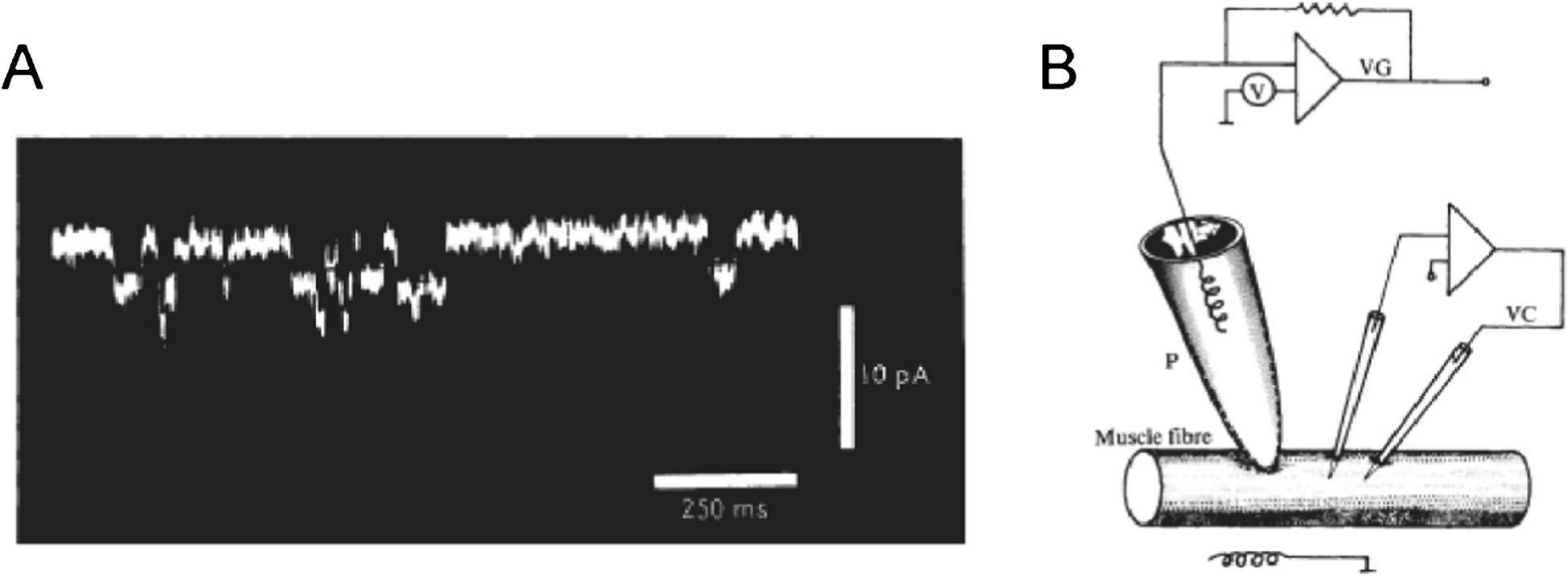
First single-channel current recording from frog muscle. **A** Oscilloscope recording of current through a patch of membrane of approximately 10 µm.^2^. Downward deflections represent inward currents. The patch pipette contained 0.2 µM suberyldicholine, an analogue of acetylcholine which induces very long-lived channel openings. Membrane potential—120 mV. **B** Schematic circuit diagram for current recording from a patch of membrane with an extracellular pipette. VC, standard two-microelectrode voltage clamp circuit to set locally the membrane potential of the fiber to a fixed value. P, fire-polished pipette, with 3–5 µm diameter opening containing the agonist. Pipette resistance was 2–5 MΩ (reproduced from Neher and Sakmann [77])

These unitary currents postulated by noise analysis were finally shown a few years later by Neher and Sakmann [77] on the membrane of frog muscle (Fig. 2A). The two German scientists had developed a technique that allowed the recording of the extremely small electrical currents (in the order of few picoamperes) that supposedly pass through single ion channels [77]. The technique is based on an incredibly simple idea. A fine glass pipette with a very small opening (in the range of micrometers) is used to make contact with and confine a microscopic area, or patch (thus the origin of the patch-clamp method), of the cell membrane that, hopefully, will contain only a single or very few active ion channels (Fig. 2B).

Neher and Sakmann’s recordings clearly showed discrete changes in current, which were expected to occur when single channels open or close (Fig. 2A). These changes in single channel current amplitudes had most of the features that had been postulated for single ion channels, including the consonance with the estimates of single-channel conductance obtained with the analysis of macroscopic current fluctuations [19, 21]. Typical channels can facilitate ion diffusion to reach fluxes of tens of millions of ions per second, rates close to the diffusion of ions in bulk solution. The development of the patch-clamp technique earned Neher and Sakmann the Nobel Prize in Physiology or Medicine in 1991, which, in the words of the Nobel Committee, “conclusively established […] that ion channels do exist and how they function.”

### Ion channels are made of proteins

There was another issue to be conclusively addressed: what were these channels made of? By the end of the 1960s, the notion of channels as holes in the membrane had considerably grown but still remained undefined in terms of structure and material. Evidence that the postulated channels shared some properties with proteins, or that they might be at least partly made of proteins, came from several lines of observation in those years. First, the action of toxins such as TTX, which was found to obey the law of mass action for a bimolecular reaction, a behavior typical of a receptor with a binding site [76]. Second, the effect of the proteolytic enzyme trypsin on the membrane excitability of the squid giant axon [89, 90], and later of pronase, which fully and selectively removed inactivation of the Na^+^ current (incidentally, this was the observation that made Armstrong think more colorfully of the ball-and-chain mechanism for Na^+^ channel inactivation process) [7, 88]. Third, studies with the protein extract known as excitability-inducing material (EIM) [73] showed that, when inserted into artificial lipid bilayers, induced elementary current events of the type expected from the recording of membrane ion channels with stochastic behavior [10, 27]. Similar results were obtained shortly thereafter with the peptide Gramicidin A, also inserted into artificial lipid bilayers [44].

All these functional studies conducted from the early 1960s have provided increasingly convincing support for the idea that Na^+^ and K^+^ ions cross the membrane through aqueous pores provided by specific protein structures called ion channels. However, the consensus in the scientific community was not complete and many still used the word “channel” as a synonym of “ion pathway” or “ion pore,” without implying a specific protein structure with a central hole for ions to pass through.

An important contribution in this regard came with the fluid mosaic model of the membrane proposed in 1972 by Singer and Nicolson. Based on freeze-fracture EM data that showed the presence of particles of variable size (50–100 Å) emerging from the fractured plane of the membrane and fluorescence studies with tagging antibodies showing that these particles did not occupy fixed positions but moved laterally in a random manner, Singer and Nicolson [102] suggested that membrane proteins did not form a homogeneous veil stretched across the membrane, as previously thought [22], but inserted themselves into the lipid matrix, sometimes crossing it completely.

While the fluid mosaic model of the membrane seemed the needed evidence to dispel the last doubts about the protein structure of the hypothetical channels, resistance to the idea that ions traveled across the membrane via pores was evidently still strong if Armstrong et al. in 1973 wrote: “The ionic channels of nerve membrane and the gates that control ion movement through them are widely supposed to be composed of protein, but there is surprisingly little evidence on the question.”

A few years later came the result that more than any other consolidated the idea that ion channels were made of proteins. Using the high-affinity and highly selective TTX for the Na^+^ channel, Agnew and coworkers [2] extracted and purified from electric eel electrocytes a protein of about 230 kDa, with an estimated Stokes radius close to 90 Å. This protein purified from TTX was subsequently found to conduct Na^+^ ions when inserted in liposomes, as visualized at the single-channel level [91].

### From functional notion to molecular entity

To summarize, the main lines of evidence that led ion channels to be accepted as protein structures that cross the entire membrane and support permeation for different ions are as follows. First, the observation that certain agents selectively blocked Na^+^ or K^+^ currents suggested the notion of ions passing through distinct pathways (channels). Second, (i) the action of toxins such as TTX, which proved to obey the law of mass action for a bimolecular reaction, a behavior typical of receptors with a binding site; (ii) the effects of proteolytic enzymes on excitability and membrane currents (in particular, the action of pronase on the inactivation of Na^+^ currents); (iii) the observation that protein extracts as EIMs, inserted into artificial lipid bilayers, induced elementary current events as expected of a membrane ion channel. Third, the EM data of freeze fracture and Singer and Nicolson’s fluid mosaic membrane model, which showed the presence of large particles within the cell membrane reaching both internal and external solutions, provided the physical ground for the protein nature of ion channels. Fourth, the square-shaped, picoampere-size currents switching on and off observed with the patch-clamp technique. Together, these observations indicate that there are separate pathways for ions and that they are made up of proteins.

**Fig. 3:**
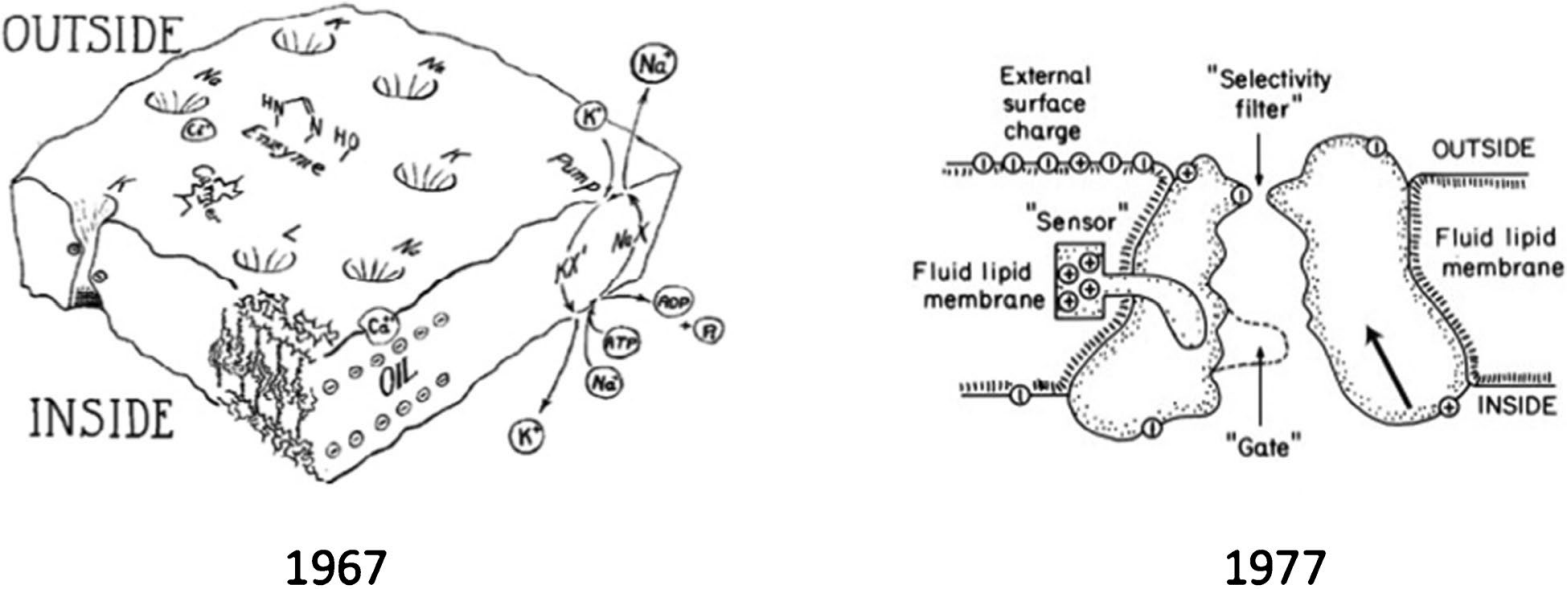
Change of vision on ion channels. Drawing from Bertil Hille illustrating the development of the ion channel concept from rather featureless ion-specific holes in a membrane model reminescent of that of Davson and Danielli with a central oily matrix and protein adsorbed peripherally to individual protein macromolecules spanning the membrane with a selectivity filter located toward the extracellular space and a voltage sensor operating as a cytoplasmic gate. Left: From Hille’s PhD thesis, 1967. Right: From Hille [39]

These observations brought even the most reluctant scientists to gradually reconsider their views on ion channels. However, the conceptual transition was a rather long process, and perhaps the decade 1967–1977, if we are to give a time frame, was critical in this respect, as well illustrated by the two cartoons in Fig. 3, drawn by Hille in 1967 for his doctoral thesis (left) and for a scientific article 10 years later (right).

**Fig. 4:**
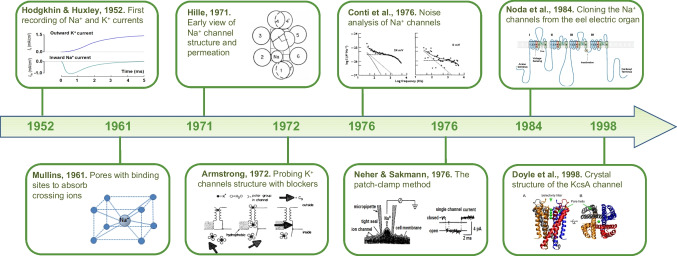
Timeline of main events that led to the demonstration of the presence of ion channels in cell membranes and the prediction of their structure

We note that the 1977 cartoon already shows all the putative structures underlying the channel’s main functions. The “sensor,” the charged structure that moves across the membrane as the voltage changes, postulated 25 years earlier by Hodgking and Huxley, and whose movement originates the gating currents first recorded by Schneider and Chandler and separately by Armstrong and Bezanilla in 1973. The “selectivity filter” underlying ion permeation, which Hille first described with Eyring’s rate theory, was based on the then-bold assumption that ions cross the membrane through channels. The “gate” controls ion flux and is positioned on the inner side of the membrane based on data obtained by Armstrong and Hille with internally applied organic and inorganic blocking ions. We conclude this section with a cartoon that recalls the key events that led to the demonstration that ion channels are the integral protein structures in cell membranes that provide the pathways for the selective, passive flow of ions [Fig. 4].

### What came next

In 1982, Numa and coworkers cloned the nicotinic acetylcholine receptor channel, nAChR, from *Torpedo californica* [81, 82] and in 1984, they cloned the voltage-gated Na^+^ channel from *Electrophorus electricus* electroplax [79]. The Na^+^ channel protein, which we have discussed at length here, was found to consist of a single polypeptide chain of over 1800 residues, which folds to form four homologous domains (DI-DIV). Based on the hydropathy analysis, each repeat was predicted to contain six transmembrane *α*-helical segments (S1 to S6). Most interestingly, one of the segments of each domain—the S4 segment—was shown to contain four to seven positively charged residues (usually arginine) systematically interposed by two uncharged residues.

This peculiar and consistent concentration of positively charged residues in the S4 segments immediately suggested that they might act as the voltage sensor of the channel, moving outwards in response to membrane depolarization, an idea that Catterall [17] and separately Guy and Seetharamulu [31] systematized in the “sliding helix”/“helical screw model.” The model suggests that positive charges, attracted by the negative internal potential, pull the S4 segment inwards in the resting state. Upon depolarization, the inward-directed forces on the S4 segment are released and the segment is pushed outwards, making the positive charges move up and interact in succession with the negatively charged residues on neighboring transmembrane segments. Shortly afterward, the Shaker K channel from *Drosophila* and Ca^2+^ channels of various muscle types were cloned and found to closely mirror the overall architecture of the Na^+^ channel, with four domains or distinct subunits forming the channel, each with six putative transmembrane segments and the S4 segment containing several positive charges interposed by two uncharged residues [67, 84, 107]. Fluorescence measurements and cysteine accessibility studies confirmed the postulated outward movement of S4 segments in response to depolarization [59, 118, 120].

#### The amino acids directly shown by mutagenesis to be the main charges in the S4 segments involved in channel activation

Electrophysiological studies on the effects of charged residue mutations within the S4 segment on the voltage dependence of Na^+^ and K^+^ channel activation have provided evidence that the positive charges of the S4 segment are at least part of the gating charge associated with channel activation [61, 97, 106]. However, these studies have not always been able to establish the quantitative contribution of each specific charge in the S4 segment to the activation charge.

This goal was pursued by similar studies on Shaker K^+^ channels, in which the effects of mutation of charged residues in the S4 segment were tested on the gating currents; these showed that the first (outermost) three/four charges are the ones crucial for channel activation. Specifically, Aggarwal and MacKinnon [1] found that only the neutralization of each of the first four positive charges (R1–R4) resulted in a substantial decrease (∼4e^–^ each) in the gating currents. This result would suggest that all four charges, from R1 to R4, of all the four subunits of the Shaker channel move all the way through the transmembrane voltage drop upon channel activation. Using a similar experimental approach on the same Shaker channel, Bezanilla’s group found the first three charged residues in the S4 segment to contribute significantly to the gating charge [98]. Together, these studies indicate that the movement of the first three/four charged residues of the S4 segment are the ones that mainly contribute to channel activation. These results were later confirmed by, among others, Elinder and coworkers who showed, based on metal-ion bridges experiments and structural information, that the S4 segment moves for about 12 Å, from the open state to the resting state; in this movement, three gating charges cross the full membrane voltage drop, or the gating pore. However, a deeper resting (closed) state can be reached with very large hyperpolarizations, which displace the S4 segment more inward and involve the crossing of more gating charge [32].

#### The linker between subunits involved inactivation of the Na^+^ channel

In the late 1970s, Armstrong and Bezanilla [6] proposed the ball-and-chain model to explain fast inactivation of the Na^+^ channel. At the turn of the 1980s and 1990s, the ball-and-chain mechanism of inactivation was shown to be valid for the Na^+^ channel. Mutational studies in rat brain Na^+^ channels showed that the cytoplasmic linker between domains III and IV (L_III–IV_) is critical for fast inactivation, possibly forming a fast inactivation particle [71, 86, 106, 114]. Specifically, a sequence of three hydrophobic amino acids, IFM, was shown to be crucial because their substitution with polar residues eliminated inactivation, suggesting that inactivation is stabilized through hydrophobic interactions [114]. The same sequence, added on both sides with a lysine to form the peptide KIFMK, was capable of inducing inactivation when applied from the cytoplasmic side [25], although with a kinetic significantly different from typical inactivation [108]. Experiments were also conducted to identify the Na^+^ channel region that would serve as the docking site for the inactivation ball, which was tentatively found in the stretch of hydrophobic residues IFMT in the linker L_III–IV_ [108, 119] and in the cytoplasmic end of the S6 segment of domain IV [63]. Over about the same years, the ball-and-chain inactivation mechanism was also shown to be valid for the Shaker channel [53, 121].

#### The identification of the amino acids lining the pore

Mutagenesis studies, the use of the pore-blocking neurotoxin TTX, and homology modeling have been crucial to understand the molecular structure of the eukaryotic Na^+^ channel selectivity filter and the outer portion of the pore. In particular, they showed that the selectivity filter is composed of the sequence-aligned residues aspartate, glutamate, lysine, and alanine, which form the DEKA ring. This structure is important for ion selectivity since replacing, for instance, lysine with glutamate abolishes Na^+^ selectivity, making the channel permeable to Na^+^, K^+^ and Ca^2+^ [85, 95]. Instead, the Na^+^ channel becomes exclusively permeant to Ca^2+^ when all DEKA residues are mutated to their counterparts in Ca^2+^ channels (EEEE) [85, 95]. A second ring is present higher up in the pore, formed by two glutamates and two aspartates (EEDD), known as the outer ring. Besides being important in Na^+^ permeation, the negatively charged amino acids of the outer ring were suggested to form the binding site for the pore-blocking neurotoxins TTX and STX, because their mutation significantly affected the binding of both neurotoxins [80, 112]. Chen and Chung [18] confirmed the interaction of the guanidinium group of TTX with the carboxylic acid side chains of the outer ring of the Nav1.4 channel by MD simulation.

Substantially different is the selectivity filter of K^+^ channels being made by a succession of four binding sites, as shown by X-ray crystallography of the bacterial KcsA channel [24] where ions pass in single file, as originally proposed by Hodgkin and Keynes [51] and Hille and Schwarz [42], and later confirmed by MD simulation [23, 30, 100]. The binding sites are formed by the backbone carbonyl groups from the conserved P-loop sequence TVGYG together with the threonine hydroxyl group [24, 125]. K^+^ permeation is essentially based on a knock-on mechanism, the only apparently capable of explaining the high throughput rate of the channel [58, 60]. According to this mechanism, K^+^ ions dwelling in the selectivity filter get a kick to move forward and eventually exit the pore from an entering K^+^. However, still discussed is the participation of water in the process, which made to distinguish a “soft” and a “hard” knock-on mechanism on whether water participates or not [68].

#### Appearance of the first crystallographic structures

At the turn of the century, MacKinnon’s laboratory began to provide X-ray crystallographic structures of ion channels from simple two-transmembrane-segment and voltage-independent bacterial channels [24]. When the first reliable voltage-gated K^+^-channel structure of the Shaker family was released (chimera Kv1.2/Kv2.1; [62]), it was found to consist of four individual subunits, each containing six transmembrane helices (S1–S6) organized to form the central pore and selectivity filter with S5 and S6 segments and the voltage sensing domain (VSD) with the positively charged S4 segment—the true voltage sensor—assembled with S1–S3 segments, located at the periphery of the channel pore module. Subsequent structures of other K^+^ channels and Na^+^ and Ca^2+^ channels have shown that they are all built on the same general architecture and share the same basic mechanistic principles [87, 99, 115–117, 123, 124].

**Fig. 5:**
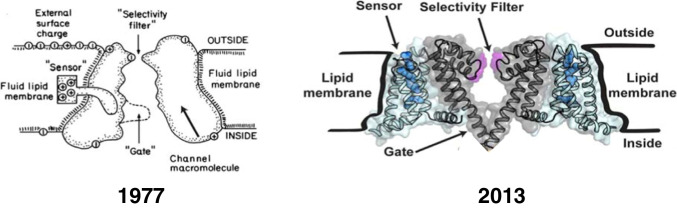
Nothing ever seems to change. Left: Cartoon of a voltage-gated channel as it could be imagined in the late 1970s (after [39]). The channel is a protein macromolecule forming a pore through the lipid-bilayer membrane. The pore has a narrow selectivity filter near the outside and a gate near the inside. A voltage sensor extending into the lipid moves under the influence of the intramembrane field and controls the opening of the gate. Dimensions and shapes of the various components are not known. Right: Structure of the bacterial Na channel BacNav. Key elements as the selectivity filter, the voltage sensor, and the internal gate are apparent and labeled. The intracellular domains extending into the cytoplasm have been removed. Arginine residues in the S4 voltage-sensor are shown as blue spheres (modified from Isacoff et al. [54])

These past few decades of intensive studies with new approaches and increasingly sophisticated techniques have undoubtedly provided a much deeper understanding of what voltage-gated channels are and how they work. Yet, looking at the cartoon model from the late 1970s (Fig. 5, left) and the crystallographic images obtained almost 40 years later (Fig. 5, right), one might get the impression that little progress has been made in these many years since the basic structures that crystallography has shown were already all there back then: the central pore, the narrow selectivity filter on the outside, the central aqueous cavity, the internal gate, and the voltage sensor with the gating charges.

The cartoon thus shows us the extraordinary insight of those scientists active in the 1960s and early 1970s, who were able, with comparatively limited resources, to identify all the main structural elements and mechanistic features of classic voltage-gated channels. This is certainly not to say that science has stood still over these years. On the contrary, enormous progress has been made in terms of the detail, scope, and impact of many aspects of ion channel structure and function, as some recent reviews for interested readers demonstrate [11, 14, 16, 68, 92].

## Conclusions

Asked why a skeptical medical student would take an interest in the study of ion channels, Clay Armstrong, upon receiving the Albert Lasker Basic Medical Research Award in November 1999, gave the following answer: “I think that ion channels are the most important single class of proteins that exist in the human body or anybody for that matter” Undoubtedly, Armstrong knows well that all proteins of the body are crucial and that we cannot do without most of them; undoubtedly, Armstrong is biased in favor of ion channels after a lifetime spent with them. Yet, if he says that ion channels are of outstanding importance, then there must be something very special around them.

One thing is certainly the predominant role they play in crucial cellular processes, such as cell excitability, signal transmission, synaptic release, muscle contraction, cell growth and death. Another is the identification of many human disorders—channelopathies—they cause or contribute to as a result of functional defects due to mutations often in a single amino acid, such as diseases of the cardiovascular system (long and short QT syndromes), the respiratory system (cystic fibrosis), the endocrine system (neonatal diabetes mellitus), the immune system (myasthenia gravis), and several forms of cancer.

There is still another reason to appreciate the importance of ion channels. The sequencing of the human genome has identified approximately 20,000 to 25,000 protein-coding genes and about 230 of these code for the pore-forming subunits of all plasma membrane ion channels, making up approximately only 1.0% of the total number of protein-coding genes of the human genome. On the contrary, due to their central physiopathological role and their privileged position, with a significant portion of the protein exposed on the outer side of the membrane, which makes them easily accessible through the extracellular fluid flow, they account for approximately 18% of the total number of currently used drugs targeting ion channels, and a significant fraction of these are listed as WHO Essential Medicines [94, 105]. We hope that the skeptical student can now be convinced.

## Data Availability

No datasets were generated or analysed during the current study.
